# Maximizing the Biochemical Resolving Power of Fluorescence Microscopy

**DOI:** 10.1371/journal.pone.0077392

**Published:** 2013-10-28

**Authors:** Alessandro Esposito, Marina Popleteeva, Ashok R. Venkitaraman

**Affiliations:** Medical Research Council Cancer Unit, University of Cambridge, Cambridge, United Kingdom; University of Milano-Bicocca, Italy

## Abstract

Most recent advances in fluorescence microscopy have focused on achieving spatial resolutions below the diffraction limit. However, the inherent capability of fluorescence microscopy to non-invasively resolve different biochemical or physical environments in biological samples has not yet been formally described, because an adequate and general theoretical framework is lacking. Here, we develop a mathematical characterization of the biochemical resolution in fluorescence detection with Fisher information analysis. To improve the precision and the resolution of quantitative imaging methods, we demonstrate strategies for the optimization of fluorescence lifetime, fluorescence anisotropy and hyperspectral detection, as well as different multi-dimensional techniques. We describe optimized imaging protocols, provide optimization algorithms and describe precision and resolving power in biochemical imaging thanks to the analysis of the general properties of Fisher information in fluorescence detection. These strategies enable the optimal use of the information content available within the limited photon-budget typically available in fluorescence microscopy. This theoretical foundation leads to a generalized strategy for the optimization of multi-dimensional optical detection, and demonstrates how the parallel detection of all properties of fluorescence can maximize the biochemical resolving power of fluorescence microscopy, an approach we term Hyper Dimensional Imaging Microscopy (HDIM). Our work provides a theoretical framework for the description of the biochemical resolution in fluorescence microscopy, irrespective of spatial resolution, and for the development of a new class of microscopes that exploit multi-parametric detection systems.

## Introduction

Fluorescence microscopy provides an invaluable tool to probe cell and tissue biochemistry. Fluorophores sensitive to the physico-chemical properties of the environment or fluorescent sensors engineered to probe biochemical reactions encode biologically relevant information into changes of their photophysical properties. The read-out of these probes is typically performed with the quantitative detection of specific photophysical properties, *e.g.* excited state lifetime, fluorescence anisotropy or emission/excitation spectra. Photon-toxicity, photo-bleaching and the need for acquisition times compatible with biological processes limits the maximum number of photons that can be collected during an experiment. This limited photon budget hinders the capability of biophysical imaging techniques such as fluorescence lifetime, anisotropy and spectral imaging to unmix complex biochemical signatures and to resolve small changes in biochemical systems. Theoretical frameworks describing the role of photon-statistics in various techniques have been developed in order to define these limits and to provide tools that may serve for the optimization of detection schemes [1]–[5].

Over the past decade, most academic and industrial developments in microscopy have focused on spatial super-resolution techniques [6], [7]; however, the capability of fluorescence microscopy to discriminate different biochemical and physic-chemical environments does not depend only on spatial resolution: detection schemes aiming to enhance biochemical/physico-chemical resolution of fluorescence microscopes are equally fundamental, in particular for full exploitation in cell biology. Intuitively, multi-parametric detection [8], [9] is an obvious strategy to achieve this goal. Indeed, various techniques developed in the past decades, *e.g.*, spectrally resolved lifetime imaging [8], time-resolved anisotropy imaging [10] and spectrally-resolved anisotropy imaging [9], already provide detailed information concerning certain parameters. Detection technologies are mature to integrate all these modalities into a single instrument. However, these multi-parametric imaging platforms are sometime met with skepticism because of the high cost and complexity of multi-parametric or multi-modal systems and the lack of a rigorous definition of “biochemical resolving power” that would permit to demonstrate advantages of these detection schemes on theoretical grounds. Here, we present a theoretical framework that generalizes Fisher information theory for a system with multiple detection channels with the aim to provide a mathematical background with which it is possible to define and to study the biochemical resolving power of an optical system as a function of photon statistic, independently from its spatial resolution. Furthermore, we describe strategies to optimize multi-dimensional detection systems, provide software for their analysis and review sources of photon-losses that may limit their efficiencies.

Fisher information theory has been conveniently used to study the upper boundary of signal-to-noise ratios (SNR) achievable in fluorescence detection [1], [5]. These works have been primarily applied to the study of single-photon counting systems because single photon counting is mostly affected by Poissonian noise. All other techniques may introduce additional types of noise at the detriment of SNR. However, at high signal-to-noise ratios and with optimized detection schemes [3], analog techniques may converge to the upper boundaries described by Fisher information potentially providing other benefits, *e.g.* higher acquisition throughputs [3]. Therefore, all results obtained with the application of the following theoretical framework should be regarded as the physical limits in the precision of a detection system and any system that would approach this limit will be defined as “efficient” [3].

Furthermore, we aim to lay down the theoretical foundations (and justifications) for multi-dimensional techniques and, more specifically, for spectrally- and polarization- resolved time-correlated single-photon counting that would enable the parallel detection of all properties of light. For conciseness, this technique will be referred to as Hyper Dimensional Imaging Microscopy or HDIM [9]. Furthermore, as phasor transformation [11] has become widely used in the analysis of biophysical imaging data, we propose a generalization of this technique to multi-dimensional datasets. We show that parallel multi-parametric imaging modalities can maximize signal-to-noise ratios and boost the resolving power of biochemical/biophysical imaging techniques. Thus, our work lays a theoretical foundation for the development of HDIM platforms, and demonstrates some of the potential advantages of such systems in resolving cell biological processes.

## Theory

In this work, Fisher information is used in order to define and describe the physical limit in biochemical resolving power of an optical system and to characterize how a multi-channel detection system can attain the highest possible biochemical resolution from a theoretical standpoint. In this section, we provide definitions and theorems that enable the description of biochemical precision and resolution in fluorescence microscopy; the demonstrations can be found in the Methods section; the validation of the theory and the description of practical tools for the analysis of optical systems can be found in the Results section.

A multi-channel optical instrument partitions detected photons into histograms of photon counts collected over ranges of arrival times, wavelengths and polarization states. It is possible to characterize the general properties of the Fisher information content provided by any given partition and to demonstrate that detection systems of higher channel number and dimensionality increase the information content of an experiment as stated in the “photon partitioning theorem” below.

***Photon Partitioning Theorem*** (See also Methods: Proof of Photon Partitioning Theorem)

Let π be a partition of *N* photons obtained with *m* independent noiseless channels resulting in the observable random variable 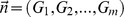, where *G_i_* is the photon-count value measured by the *i*-th channel. Let 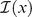 be the Fisher information on the unknown parameter *x*. If π’ is a finer partition of the *N* photons obtained with *m*’>*m* independent noiseless channels then 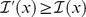.

In analogy to literature related to information theory [12], a noiseless channel is defined as a channel that does not introduce errors/noise. In the context of fluorescence, a noiseless channel reveals shot noise that is caused by the inherently Poissonian process of photon detection. This is not only a useful and common abstraction, but it is representative of single photon counting detectors. The random variable *x* may represent physical quantities such as the fluorescence lifetime of a fluorophore, FRET efficiency or fluorescence anisotropy, or a biochemical quantity such as pH, a fraction of bound molecules or a relative enzymatic activity.

Although for a finer partition of photons the Fisher information may increase, the trivial duplication of a detection channel that collects light on an identical spectral, polarization and time range, cannot provide any advantage, but may rather increase only photon-losses or noise (see practical non-trivial partitioning note and Text S1 on costs and photo-losses). The following corollary and note clarifies that maximal Fisher information can be attained 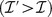 solely if the partitioning of photons is carried out on partitions that maximize the gradient of the partitioning function relative to the parameter of interest.

***Non-trivial Partitioning Corollary*** (See also Methods: Proof of Non-trivial Partitioning Corollary)

For any given detection channel with photon counts *G_i_* there is a partitioning function 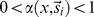 such that the two newly defined detection channels will detect 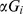 and 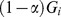 photons, respectively. 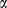 is a function of the unknown parameter *x* and of the (hyper-dimensional) spectral properties 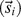 of each detection channel. If the necessary and sufficient condition 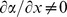 is satisfied, the new partition provides a net increase 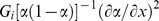 of Fisher information 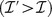 and 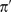 will be called “non-trivial”.

Here, the vector 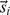 defines the boundaries of each detection channel including the band of observed wavelengths, the orientation of an analyzer and the time windows of photon arrival times that is considered in the photon counting process (see also Proof of Photon Partitioning Theorem). An example of partitioning function for the case of fluorescence lifetime imaging is described in the *Methods – Partitioning function: an example*.

A fundamental and direct outcome of the photon partitioning theorem is that multi-channel multi-parametric detection systems are more photon-efficient (the capability to approach relative errors limited just by Poissonian noise) [2], [3].

***Photon-efficiency Corollary*** (See Methods: Proof of Photon-efficiency Corollary)

For a non-trivial partitioning of *N* photons onto *m* detection channels, the variance *σ_x_^2^* of an unbiased estimator of the parameter *x* will asymptotically decrease with *m* increasing, *i.e.* the photon-efficiency of a measurement increases with non-trivial increments of detection channel number.

Photon-efficiency can be described by the figure-of-merit “*F*” (or F-value) introduced by Gerritsen *et al.* [2] for FLIM that can be easily generalized to 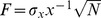, *i.e.* the ratio between the relative error 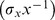 that affects the estimate of *x* compared to Poissonian noise 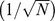. An F-value of 1 signifies that the estimate of *x* is affected only by Poissonian noise (it is efficient) and for an F-value of 2, the estimate of *x* exhibits twice the noise compared to a shot-limited measurement as if a fraction equal to 1-2^−1/2^ of the detected photons were lost. Therefore, a measure of photon-efficiency is 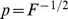 [3] (for an analysis of actual optical losses see **Text S1 - Costs and photon-losses: case studies** and the *Practical non-trivial partitioning note*). The increased precision in the detection of photophysical/biochemical quantities results in enhanced biochemical resolving power as stated below by the separability and resolving power corollary.

***Separability and Resolving Power Corollary*** (See Methods: Proof of Separability and Resolving Power Corollary)

Let consider a photo-physical system that is sensitive to physico-chemical (biochemical) properties of its environment. If *E_1_* and *E_2_* are two different physico-chemical environments characterized by the parameter *x* and *distance* described by 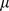 = |*x_1_*−*x_2_*| then, for a non-trivial partitioning of *N* photons onto *m* detection channels, the separability 
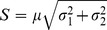
 of the detection system will asymptotically increase with *m* increasing. *Separability* [1] is a less ambiguous (statistical) definition of resolving power; however, it is possible to define biochemical resolving power also applying the Rayleigh criterion (*i.e.*, *S*∼2) in analogy to the description of spatial resolution. The smallest resolvable difference between *E_1_* and *E_2_* at *x = x_0_* (Δx, *i.e.*, the biochemical resolution) can be described thus by 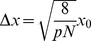 and, therefore, the *biochemical resolving power* is 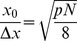. Therefore, at increasing number of channels, the photon-efficiency *p* of a fluorescence microscope increases together with its biochemical resolving power.

This work is concerned with the biochemical resolution of a microscope; however, when *x* is a photophysical property (*e.g.*, the fluorescence lifetime of a fluorophore) a consequence of the photon partitioning theorem is that the photophysical resolving power of a fluorescence microscope increases for *m* increasing. Therefore, this definition is a generalized description of resolution useful to define how small photophysical or biochemical quantities can be resolved either within a pixel of an image or between two different areas of a sample. The definitions of separability, resolution and resolving power as affected by the instrument response function can be found in Methods – Proof of Separability and Resolving Power Corollary.

### Practical Non-trivial Partitioning Note

The photon partitioning theorem and its corollaries were described in the case of an ideal system in which the addition of a detection channel can be done without any penalty. This is useful to define the physical limits in Fisher information, precision and biochemical resolution of an optical system and not just the limit imposed by current technologies. In reality, the addition of detection channels can result in photon-losses (*i.e.*, partial loss of information) or more general constrains or costs (*e.g.*, the actual cost of an optical system). If these generalized cost 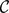 can be quantified, a non-trivial partitioning will be practically achievable if and only if 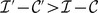. The analysis of photon-losses caused by optical relay systems (see **Text S1– Costs and photon-losses: case studies**) or additional sources of noise (see **Methods –** **Numerical optimization of Fisher information**) is therefore fundamental for the realization of the physical limits of the biochemical resolving power of a fluorescence microscope.

## Results

### Numerical Optimization of Fisher Information

The non-trivial partitioning of photons with higher channel density increases the physico-chemical (or biochemical) resolution of a detection system. Practical implications of the photon partitioning theorem can be demonstrated by implementing numerical optimization of a detection system. Fast iterative algorithms can identify the best number and configuration of detection channels by the numerical maximization of 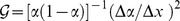, a factor that is directly proportional to the absolute gain in Fisher information (see *Non-trivial Partitioning Corollary*; Δ indicates numerical estimations of derivatives). A detection channel is split into two new detection channels only when 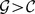, where 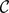 represent the cost of adding a new detection channel. The physical meaning of 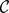 can be, for instance, a photo-loss (see also Supporting Material – Costs and photon-losses: case studies) or additional noise (see Methods – *numerical optimization of Fisher information*); however, this cost term can be used as a mere numerical tolerance for the iterative algorithm. Similar algorithms can be implemented by evaluating the loss 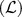 of Fisher information when two channels are merged into one. The optimal partitions 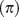 obtained with these iterative strategies 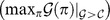 or 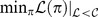 can be further optimized by the numerical minimization 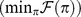 of the F-value 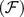 [2], [3], [13], *i.e.* the ratio between the relative error 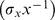 that affects the estimate *x* compared to Poissonian noise 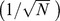: 
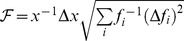
, where *f_i_ = G_i_/N*. 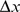 is a small numerical value, for instance in the order of a few percent or a few thousands of *x* used for the numerical evaluation of 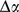 and 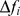 (see Methods – *numerical optimization of Fisher information* and Supporting Material – *Matlab code for Fisher information analysis*).

### Optimization of Time Gating

Fluorescence lifetime imaging microscopy can be accomplished with a number of detection schemes, in the time- or frequency- domain and with single photon counting or analog detection [14]–[17]. For simplicity, in this work we consider only time-correlated single photon counting (TCSPC) [18], [19] and time-gating [20] where photon arrival times relative to repetitive pulsed excitation are measured and histrogrammed in a number of time bins. The precision of these time-domain techniques as a function of instrument parameters (*e.g.*, laser repetition period *T*, number and shape of time bins), fluorescence lifetime (*τ*) and photon count per pixel (*N*) has been thoroughly described [1], [2], [21]–[23]. Therefore, it is instructive to compare results inferred from the general properties of Fisher information relative to state-of-the-art optimization techniques. First, we describe how to optimize the position of time gates by analyzing the simple case of detection based on just two time gates; second, we generalize this optimization strategy to detection systems with multiple gates and TCSPC.

**Figure 1:**
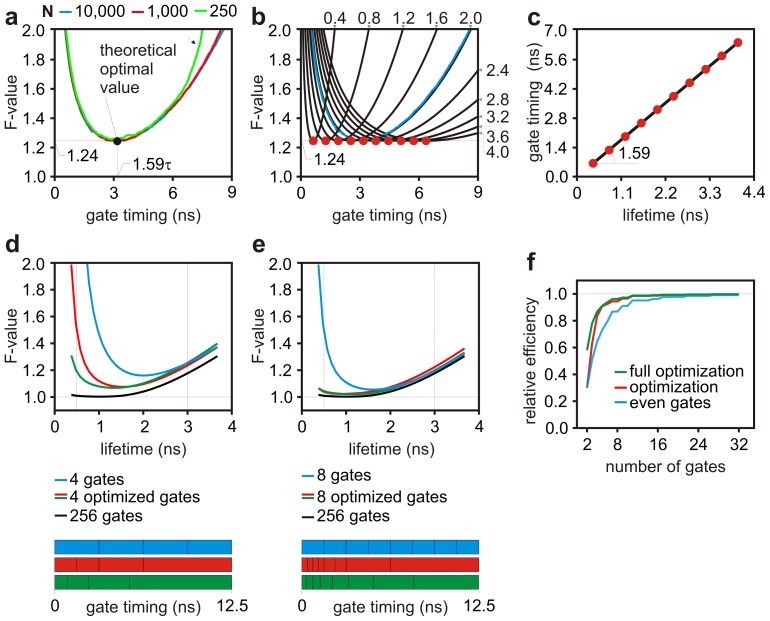
Optimization of time gates. A set of three typical Monte Carlo simulations (**a**) ran with different photon counts (cyan: 10,000, red: 1,000 and green 250) to evaluate the performance of the system (F-value) as a function of the position of a time gate for a two gate lifetime detection system. The minima of the curves (the best performance) match the theoretical values. At low photon counts (green curve and arrow) the results deviate by the others for high values of gate timings because of the presence of bias caused by too low photon counts in the second gate. The F-value is also estimated numerically by the direct estimation of Fisher information (**b**). Here the blue curve is the same of panel **a** and it is plotted for reference. **c**) The best position of the time gates (red circles in **b** and **c**) is also plotted as a function of the simulated fluorescence lifetimes (from 0.4 to 4.0 ns). The slope of the line identifies the best position of the second gate matching the predictions illustrated in panel **a**. Numerical optimization of Fisher information for a system with 4 (**d**) and 8 (**e**) gates are also shown; the 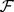-value for 4 or 8 gates is plotted for gates of equal width (blue), for gates optimized by 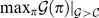 (red) followed by 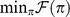 (green) and for a reference partition over 256 time gates (black, representative of TCSPC). The vertical lines delimit the regions within which the optimization algorithms were used. The time gating scheme is represented by the boxes at the bottom of panels **d** and **e**. The efficiency of the optimization strategies relative to TCSPC 
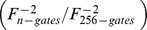
 is also plotted *versus* the number of gates (**f**) for gates of equal width (blue, “even gates”), for gates optimized by 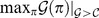 (red, “optimization”) and then followed by 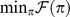 (green, “full optimization”).

Gerritsen *et al.* have shown that time gates (*channels*) of uneven width can significantly improve the precision of fluorescence lifetime imaging microscopy compared to a system that provides the same number of time gates but with uniform width. The demonstration that time gates of uneven width increase the F-value in FLIM was obtained with Monte Carlo simulations over a number of test partitions because analytical solutions are often difficult or impossible to obtain [2]. These results have been confirmed also by the analytical description of the F-value that has been recently evaluated for two gates of uneven width [13]. Figure 1a shows the results of Monte Carlo simulations ran to identify the time (*ρτ*) when the second time gate should start provided that this time gate will close (at time *T*) before the next laser pulse. The optimal value of *ρ* that minimizes the F-value (∼1.24) is ∼1.58 (*e.g.*, for a lifetime of 2 ns, the second time gate should start at 3.16 ns). These values are practically identical to those found analytically (F_best_ = 1.25, *ρ* = 1.59; see Methods – *Optimal gates for two time gates system* for details and Fig. 1a, black circle) [13].

The *photon partitioning theorem*, however, permits to solve optimization problems just by the analysis of the general properties of Fisher information without requiring laborious Monte Carlo simulations or analytical solutions. Figure 1b and c show the F-value curves obtained by the simple numerical maximization of the gain function 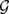 and the linear regression between the estimated best value of *ρτ* and the simulated value *τ*, respectively. These data permit to establish that the best F-value achievable for any value of fluorescence lifetime is 1.24 and that the best position of the start of the second time gate is at 1.59 times *τ*. Notably, the numerical maximization of the Fisher information gain provides, in less than 2 ms per tested lifetime, the very same results obtained with lengthy Monte Carlo simulations (∼3hours per simulated SNR) or with laborious evaluations of an analytical solution. Moreover, analytical solutions to find optimal gating with multiple time gates are unlikely to exist; however, a simple optimization algorithm based on the *non-trivial Partitioning Corollary* can find solutions very efficiently (<20 ms). Figure 1d–e shows optimization of gating strategies performed with either 4 or 8 gates, the number of gates often used in time-gating. The blue curves show the performance of a standard even partition of the period *T* in 4 (Fig. 1d) and 8 (Fig. 1e) gates and the black curves demark the best performance that can be achieved by TCPSC (*e.g.*, with a full bin resolution on the arrival times histogram equal to 256, method equivalent to detect photon counts onto 256 adjacent time gates of equal width). Red and green curves show the F-value obtained by time-gates that are iteratively optimized with 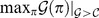 and 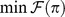 (optimization software is provided in Supporting Material). Importantly, the gain of Fisher information is optimized over a number of fluorescence lifetime values at the same time (from 0.5 to 3.0 ns) permitting to obtain an optimal response over a very broad range of possible experimental conditions. Figure 1f shows the efficiency of each gating scheme relative to TCSPC as expressed by the average value of the ratio 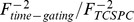 evaluated over the 0.5–3.0 ns range of fluorescence lifetime values. A relative efficiency of 75% means that the precision of the system is as if 25% of photons are lost and, therefore, the system will have to increase the acquisition time of 0.75^−1^ times in order to recover the same SNR compared to TCSPC. Therefore, the numerical analysis of Fisher information theory permit to evaluate which is the best performing and simplest detection system that should be used for FLIM.

### Optimization of Spectral Channels

Spectral detection is the detection of fluorescence over a number of spectral channels. Detection can be achieved by *i*) sequential imaging of fluorescence emission over a number of spectral bands selected with filters or other devices (spectral imaging), *ii*) simultaneous detection of fluorescence emitted over a few spectral bands with the use of a number of photodetectors (multi-colour or multi-spectral imaging) or *iii*) simultaneous acquisition of spectral information using a spectrograph and an array of detectors (hyper-spectral imaging). Nowadays, spectral, multi-spectral and hyper-spectral imaging techniques are commercially available with high-end laser scanning microscopes and commonly used for spectral unmixing of many fluorophores. Neher and Neher characterized the role of photon-statistics in spectral imaging and discussed the Rao-Cramer limit that can be achieved with two spectral channels [24]. At the best of our knowledge, there are no other works that describe the role of photon-statistics in spectral imaging for applications to fluorescence detection.

**Figure 2:**
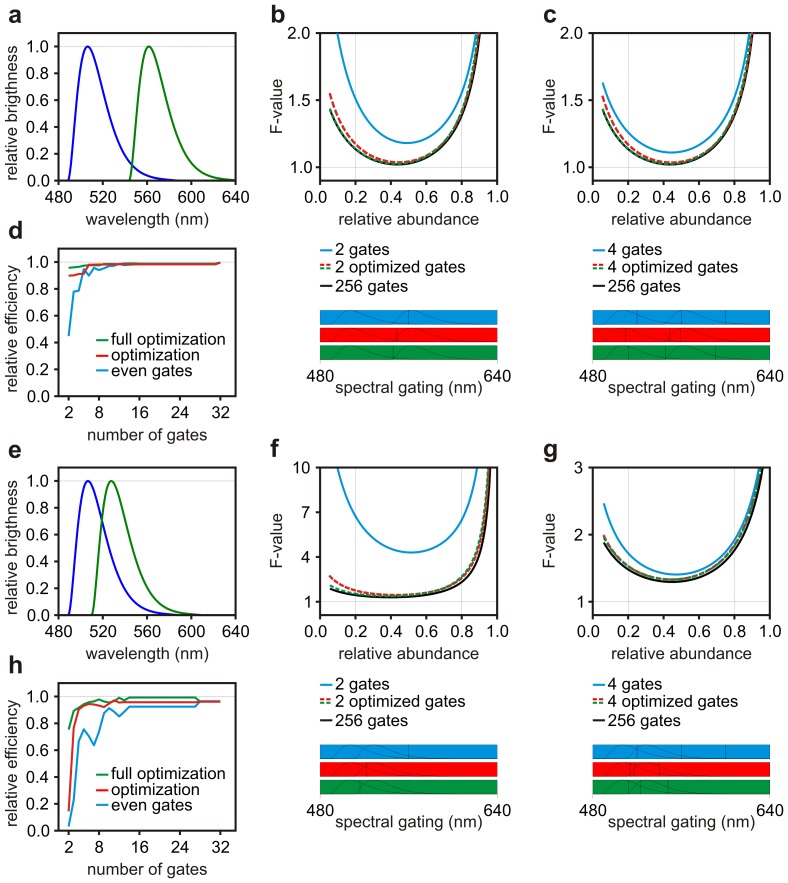
Optimization of spectral channels. Spectra representative of fluorophores like EGFP and mCherry (**a**) or EGFP and EYFP (**e**) were used to carry a numerical optimization of the spectral gating scheme as a function of the relative abundance of EGFP. Results are shown for a a system with 2 (**b** and **f**) and 4 (**c** and **g**) adjacent spectral channels which relative positions were set equal (blue) or optimized numerically by 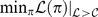 (red) and followed by 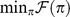 (green) with optimization carried over the relative abundance range indicated by the vertical lines. The black curves represent performance that could be achieved with hyperspectral detection over 256 spectral gates. The spectral gating scheme is represented by the boxes at the bottom of panels **b**, **c**, **f** and **g**. The efficiency of the optimization strategies relative to hyperspectral detection achieved with 256 gates 
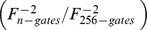
 is also plotted *versus* the number of gates (**d** and **h**).

Figure 2a shows typical spectra representative of fluorophores like EGFP and mCherry; these spectra were used to test algorithms developed to optimize the performance of spectral imaging. Optimization of spectral gating schemes (the assignment of a specific spectral band to a detection channel) was performed with 2 or 4 spectral gates and the resulting F-values (see Eq. (21) in Methods) are shown in Fig. 2b and c, respectively. With well separated spectra, spectral unmixing can achieve very high precision (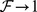) for a broad range of relative abundances. The best partitions found with 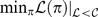 (red curve) and followed by 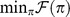 (green curve) are practically matching a spectral system providing 256 spectral bins. This is also confirmed by the relative efficiency of partitions of increasing channel number 
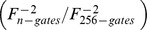
 shown in Fig. 2d. With fluorophores exhibiting much more overlapping spectra, *e.g.* EGFP and EYFP, spectral unmixing deteriorates its performance, but optimization of spectral channels permit to recover very high efficiency with few (2–4) spectral channels (Fig. 2e–h).

**Figure 3:**
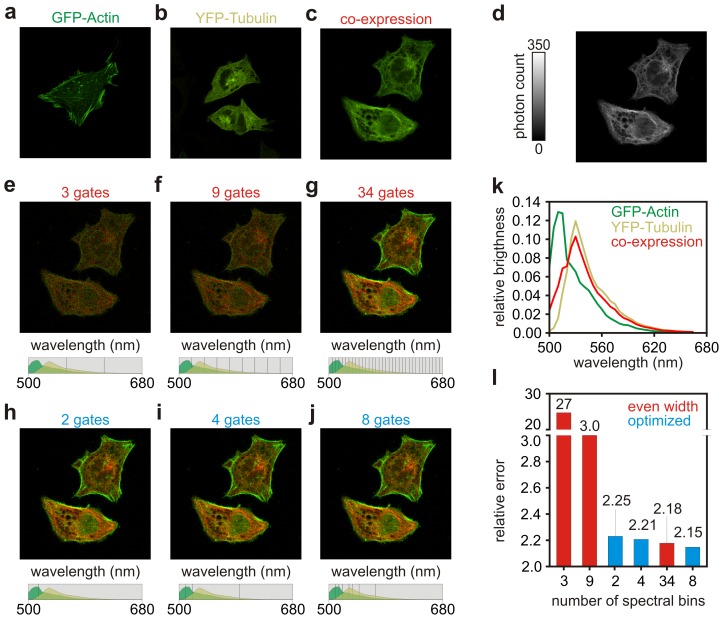
Spectral unmixing of fluorescent proteins. True color images of cells expressing EGFP-Actin (**a**), EYFP-Tubulin (**b**), both proteins (**c**) and total photon count per pixel (**d**) for the latter. Spectral unmixing was used for different gating schemes (**e**–**j**) for gates of even width with 3 (**e**), 9 (**f**) and 34 (**g**) gates and for optimized gates of 2 (**h**), 4 (**i**) and 8 (**j**) gates. The spectra gating scheme is displayed at the bottom of the panels with the reference spectra (**k**) overlaid for comparison. In green the signal recovered for GFP-Actin and in red the signal estimated for YFP-Tubulin. The comparison of the best achievable F-values measured experimentally demonstrate the positive impact of gating optimization and the trend to provide more precise results at increasing channel number (**l**).

Figure 3a–c shows true color images, *i.e.* red-green-blue representations of how the sample would look like if seen at the naked eye [9], [25], of HeLa cells expressing EGFP-Actin (a), EYFP-Tubulin (b) or both tagged cytoskeletal proteins (c). The total photon count for the latter typical measurement is also shown in Fig. 3d amounting to a few hundreds photons per pixel. The results of spectral unmixing for a number of spectral gating strategies with gates of equal width and the reference spectra of the samples are shown in Fig. 3e–k. Figure 3h–j show images obtained on the same data, but with spectral data binned together accordingly to gating strategies with gates of uneven width optimized by Fisher information analysis. The relative abundance of actin and tubulin are shown in the green and red channel, respectively, in a RGB overlay demonstrating good separation of these two fluorophores. The images demonstrate improvement in signal-to-noise ratios at increasing channel number (Fig. 3e–g) and higher signal-to-noise ratio for all optimized detection schemes (Fig. 3h–j). F-values were evaluated experimentally by repeating the acquisition of the spectral data five times and analyzing it independently in order to obtain standard deviations and F-values on a pixel by pixel basis. The distribution of the F-values are shown in Fig. S1 and the best F-values are shown in Fig. 3l confirming better results for optimized gating strategies and trends towards improved precision for higher number of detection channels.

### The Photon Partitioning Theorem and Multi-parametric Detection

**Figure 4:**
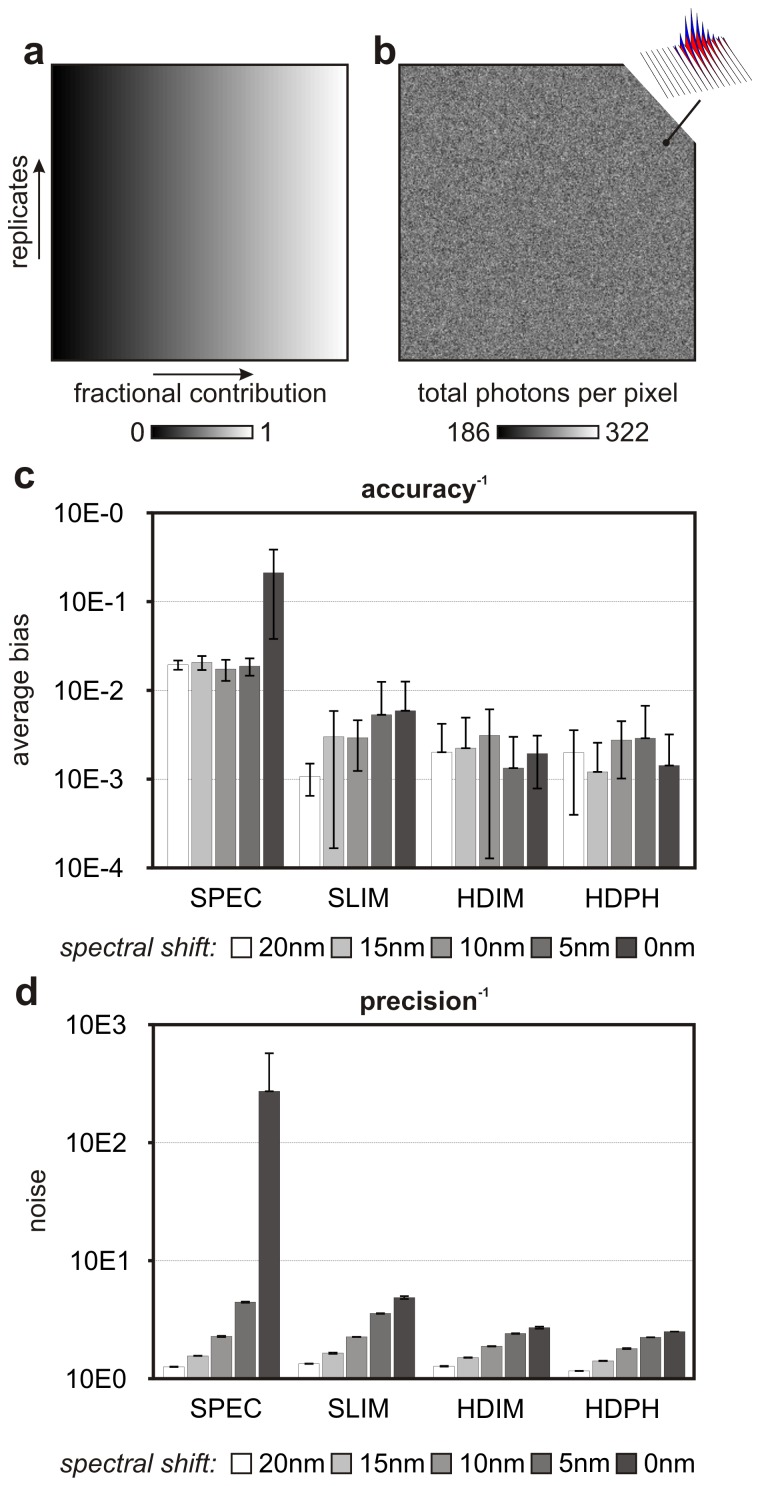
Accuracy and precision of unmixing based on hyper-spectral imaging (SPEC), spectrally resolved FLIM (SLIM) and HDIM. HDIM data was also analyzed with a generalized phasor transform (Hyper-dimensional Phasors, or HDPH). A linear gradient (**a**) of relative fractional contribution of two fluorophores was used to mix the optical signatures shown in Fig. S1 and illustrated in the inset of panel **b**. Simulations were carried out in the presence of Poissonian noise with expected count per pixel equal to 250 (shown in **b**), 1,000 and 10,000 photons. The difference between the simulated fractional contributions and the values estimated by spectral unmixing was used to compute the bias of various techniques (**c**). The vertical direction of these synthetic images was used to estimate statistical errors caused by Poissonian noise and, therefore, the precision of the analysis techniques (**d**). Accuracy and precision are shown for fluorophores with spectral peaks at various spectral shifts from each other (0 nm, 5 nm, 10 nm, 15 nm and 20 nm) around the central wavelength of 550 nm. The error bars in panel (**c**–**d**) show standard deviations computed across simulations at different noise photon count levels.

The photon partitioning theorem and its corollaries permit us to predict that multi-parametric detection schemes that exploit the various properties of light can attain higher signal to noise ratios compared to detection schemes based on fewer detection channels. This prediction was tested with Monte Carlo simulations for hyper-spectral imaging (SPEC), for the combined detection of photon arrival times and wavelength that can be achieved by spectrally resolved FLIM [26] (SLIM) and for the simultaneous resolution of photon arrival times, wavelength and polarization state that can be achieved by Hyper Dimensional Imaging Microscopy (HDIM). Monte Carlo simulations (see Methods for details) were carried out with fluorophores exhibiting typical fluorescence lifetimes (3.0 ns and 2.0 ns), intrinsic anisotropy (0.4 and 0.2), rotational correlation times (12 ns and 1.0 ns) and spectral width (50 nm); simulations were performed with varying difference between the spectral peaks of the two fluorophores (from 0 nm to 20 nm) and central wavelength set to 550 nm. The resulting Hyper Dimensional Spectral Signatures (HDSS) of the two fluorophores, *i.e.* the spectrally and polarization resolved decays, are shown in Fig. S2 (see also inset in Fig. 4b). Synthetic images of 256 by 256 pixels were generated with photons histogrammed on 2,048 channels: 2 polarization states (parallel/orthogonal to excitation light), 16 spectral bins of equal width covering the 440–630 nm range and 64 time gates of equal width spanning 12.5 ns. These acquisition parameters were chosen to match the properties of existing detection systems (see Methods for details). Figure 4a illustrates how one axis of the synthetic images was used to generate a linear gradient of the fractional abundances of the two fluorophores, whilst the second axis was used to generate 256 replicates that were used to estimate the precision of the unmixing algorithms. Synthetic HDIM images were then generated by using this abundance matrix and then adding Poissonian noise in each simulated detection channel (250, 1000 and 10000 photons per pixel) in order to test the robustness of the analysis at low, typical and very high photon count levels. Figure 4b show the resulting intensity image in the presence of Poisonian noise for the case where an average of 250 photons per pixel was simulated. In order to compare analysis of HDIM data with analyses of datasets representative of spectrally resolved lifetime imaging and spectral imaging, photon counts from the HDIM datasets were summed along the polarization and time dimensions to generate appropriate datasets of lower dimensionalities.

HDIM data analysis was carried out both over all 2,048 detection channels per pixel and with a data reduction algorithm based on a generalization of phasor-based data analysis. For this type of analysis, discrete cosine (DCT) and sine (DST) transformations were computed over only 2 spectral bands (440–540 nm and 540–630 nm) similarly to Digman *et al.* [11], but for both polarization states. DCT and DST were also computed along the spectral dimension similarly to Fereidouni *et al.* [27]. Furthermore, we generalized the phasor approach to multi-dimensional data by applying two-dimensional DCT/DST along the combined spectral/time dimension. This strategy resulted in Hyper Dimensional Phasors (HDPH) that reduced HDIM dimensionality from 2,048 to 16 (see Text S3– Hyper Dimensional Phasors (HDPH)).

Data analysis was then carried out by spectral un-mixing. The spectral un-mixing problem is described by Eq. (1) for the case of an HDIM image (*iHDIM*) with abundances matrix *A* and the matrix containing the endmembers signatures *emHDSS*:
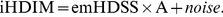
(1)


Similar equations can be written for SLIM, SPEC, and HDPH. By the inversion of Eq. (1) in the presence of Poissonian noise (250, 1000 and 10000 photons per pixel), we estimated the accuracy and precision of these various techniques (see Methods).

Figure 4c and d demonstrates the growing accuracy (lower bias) and precision (lower noise) in the estimations of the abundances of the two fluorophores obtained with Methods of increasing dimensionality (SPEC, SLIM and HDIM/HDPH) at decreasing spectral shift between two fluorophores. Bias was estimated by the average difference between the simulated abundances and their estimated values and noise was estimated by the spatially averaged standard deviations computed over the 256 simulation repeats and normalized by Poissonian noise 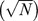. The error bars represent standard deviations across repeats at different Poissonian noise levels (details on the definition of these figures of merits can be found in Methods – *figures of merit for accuracy and precision*).

The validity of the photon partition theorem is illustrated by the increasing precision at higher channel number. The addition of detection channels along gradients established by the random variables the experiment attempt to estimate increases the information content of the measurements; therefore, the acquisition with a higher number of detection channels results in better signal to noise ratios.

### Biochemical Resolving Power and Fisher Information

**Figure 5:**
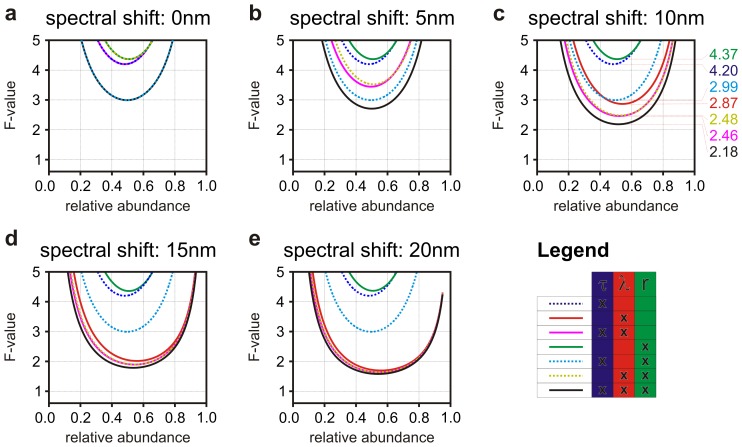
Biochemical resolving power. Panels **a**–**e** show the numerical estimations of the F-value of the same optical signatures used in Fig. 4 at increasing spectral shift for FLIM (dashed blue curve), spectral imaging (red), anisotropy imaging (green), SLIM (fuchsia), time resolved anisotropy (dashed cyan curve), spectrally resolved anisotropy (dashed yellow curve) and HDIM (black). Spectral imaging does not achieve F-values in the regions plotted in panel **a** and **b** because of the small spectral shifts considered (0 nm and 5 nm, respectively). Panel **c** shows also the minimum 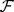-values for each technique: *e.g.*, with ∼2.18, HDIM unmixed the signatures of the two fluorophores with a relative error of ∼5% if at least 1,900 photons/pixel are collected. Spectral imaging alone requires ∼(2.87/2.18)^2^ times more photons (∼3,300) to achieve the same result.

We have shown that increasing the number of detection channels also across different photophysical parameters results in increased precision. Unmixing was used to demonstrate the possibility to separate the contribution of two different characteristic HDSS representative of two fluorophores or two biochemical/photophysical environments. In analogy to the spatial resolution in fluorescence microscopy, the resolution of biochemical techniques is also limited by photon-statistics. We developed a simple algorithm (see Supporting Material) that can determine the 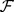-value of unmixing for all possible detection modalities. Figure 5a–e shows the results of this analysis applied to the same HDSS used to generate Fig. 4. Figure 5c shows also the best 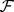-value for anisotropy imaging (4.37, green curve), FLIM (4.20, dashed blue curve), time resolved anisotropy (2.99, dashed cyan curve), spectral imaging (2.87, red curve), spectrally resolved anisotropy (2.48, dashed yellow curve), SLIM (2.46, fuchsia curve) and HDIM (2.18, black curve). How many photons are required to resolve two different biochemical/photophysical signatures? If unmixing could achieve the ideal singal-to-noise ratio limited by Poissonian noise, 400 photons would be sufficient to estimate fluorophore abundances with 5% relative error 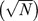. For the case shown in Fig. 5c, the 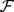-value of FLIM at a relative abundance ∼50% is ∼4.2. Therefore, the number of photons needed to resolve the mixture of the two fluorophores is ∼4.2^2^·400∼7,100. In the same conditions, spectral imaging resolves the mixture with ∼3,300 photons and HDIM, making use of all available information, with just 1,900 photons. Although we have demonstrated that increasing the number of channels always increases the biochemical resolving power in fluorescence microscopy, there are instances where simpler techniques can achieve optimal results already. For instance, if two biochemical environments exhibit a spectral shift of 20 nm, spectral imaging (Fig. 5e, 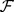-value ∼ 1.71) resolves these two environments just with ∼1,200 photons.

Therefore, the numerical analysis of Fisher information and the study of its general properties provide a useful tool to maximize the biochemical resolving power in fluorescence microscopy and to select the best (and simplest) detection system to achieve this limit efficiently.

## Discussion

In this work, we demonstrate and exemplify the “*photon partitioning theorem*” as a tool necessary to define and describe the biochemical resolving power in fluorescence microscopy/spectroscopy. The photon partitioning theorem and its corollaries permit us to generalize the analysis of photon-statistics in fluorescence detection and to demonstrate the gain in Fisher information that can be attained theoretically. This provides theoretical tools of significant practical importance (*e.g.*, the definition and characterization of biochemical resolution and methods to optimize detection systems) and also resolves a common misconception: as several hundreds or thousands of photons are necessary to fit even a simple fluorescence lifetime decay [1], the acquisition of data on separate channels has often been considered unnecessary. Moreover, the detection of many features of light is sometimes even considered impossible because of the long acquisition and analysis times that may be required. These statements hold true only if additional detection channels are acquired sequentially (as in a multi-modal microscope) or if each detection channel is analyzed independently from one another. However, whenever acquisition channels are operated in parallel [8],[9] data can be acquired at the same speed (or faster) because photons are not lost through the use of filters or analyzers (see Text S1- Costs and photon-losses: case studies). Furthermore, a net gain in Fisher information results in higher signal-to-noise ratios whenever data are not analyzed channel-by-channel but in global approaches [28], [29], as implicitly done in the applications shown in this work.

We have dedicated sections of this work to the description of practical constrains on achieving the theoretical maxima in Fisher information. In practice, the addition of a detection channel is carried out at a certain “cost”. This cost can be represented by photon-losses in the coupling optics, design complexity, additional read-out noise, or even budget. However, it is important to distinguish between the theoretical limits imposed by physics (described here) and the practical limitations of state-of-the-art technologies. Current HDIM detection systems do suffer losses, for instance, from the coupling and from the masks between adjacent anodes. However, single photon detection [30], new designs of dispersive optics [9] and novel solid state detectors [31], [32] – once integrated – will be capable of achieving or approaching the theoretical limits described by the *photon partitioning theorem*.

In this work, we have been concerned with the description of theoretical aspects of multi-channel and multi-parametric detection and with the validation of the theory by Monte Carlo simulations. Furthermore, we have demonstrated the practical application of the theory for the particular case of spectral imaging, a technique nowadays available and used in many laboratories. In order to make the advantages of these theoretical frameworks accessible, software can be found in the supporting information. These algorithms can be used to find the minimal number of detection channels that optimize fluorescence detection for FLIM or spectral imaging. Furthermore, provided that HDSSs are known experimentally or can be modeled theoretically, the best detection scheme including spectral, lifetime and anisotropy resolution can be identified. In general, Hyper-dimensional imaging microscopy will achieve the best results; however, whenever the gradient 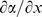 is significantly steeper along one photophysical property (*e.g.*, fluorescence lifetime, emission spectrum or polarization), simpler detection techniques may achieve or approximate the theoretical best performances. We note that fluorescence detection can be achieved with sequential excitation at different wavelengths [9], [33]; although this case was not treated explicitly in this work, sequential measurements with excitation at a different wavelength can be regarded as independent detection channels and, therefore, the theory and software presented can be applied also on this type of data.

## Conclusions

Over the last decade, much emphasis has been placed on methodologies and technologies that increase the spatial resolution of fluorescence microscopy. With this work, we aim to highlight that affordable implementations of novel detection schemes can boost the resolving power of modern microscopes in terms of physico-chemical or biochemical detection as well. The increased resolving power and capability to discern biochemical environments with spatio-temporal resolution together promise fundamental tools for applications in biophotonics, biophysics, systems biology and biomedical research.

## Methods

### Sample Preparation and Imaging

HeLa cells were transfected with EGFP-actin and EYFP-tubulin with Effectene (Qiagen) according to the protocol provided by the suppliers. Cells were fixed after 24–48 hrs with 5% PFA and kept in PBS. Spectral imaging was performed with a Leica SP5 equipped with a hybrid PMT operated in single photon counting mode.

### Simulations

Simulations were performed with Matlab (MathWorks, Cambridge, UK) on a workstation equipped with an Intel Xeon X5647 CPU and 24 GB of RAM. Images of 256 by 256 pixels were generated with photons partitioned (*i.e.*, histogrammed) on 2,048 channels. Synthetic images included 64 time gates spanning 12.5 ns, 16 spectral windows covering 440–630 nm and 2 polarization states (parallel/orthogonal to excitation light). These values were selected to match the properties of HDIM datasets that can be acquired with commercially available instrumentation (*e.g.*, two spectral FLIM systems by Becker and Hickl GmbH (Berlin, DL) coupled with a polarizer beam splitter, unpublished data). HDSSs were modeled with gamma functions and polarization-dependent exponential decays [17] as shown in Fig. S2. Synthetic HDIM images were then generated by using the abundance matrix shown in Fig. 4 and then adding Poissonian noise in each simulated detection channel (250, 1000 and 10000 photons per pixel). One simulation was carried out with both fluorophores exhibiting spectral full width at half maximum of 50 nm and peak wavelength of 500 nm. The other photophysical properties of the two simulated fluorophores were: fluorescence lifetime of 3.0 ns and 2.0 ns, rotational correlation time of 12.0 ns and 1.0 ns, and limiting anisotropy of 0.4 and 0.2, respectively. Subsequent simulations were carried out keeping all spectral properties of the two fluorophores unaltered but shifting their peak wavelengths of 2.5 nm, 5.0 nm, 7.5 nm and 10.0 nm around the central wavelength of 500 nm, towards lower and higher wavelengths, respectively. Spectral and SLIM images were generated simply by binning all photons along the time and polarization dimensions and only the polarization dimension, respectively.

The typical solution of the unmixing problem (see Eq. (1)) was achieved by unconstrained least squares and non-negative least squares, although at a higher dimensionality compared to hyper-spectral imaging. Results from unconstrained least squares are shown because the latter did not show better performances on synthetic data. In Eq. (1), *iHDIM* is a 2,048×65,563 (channel number × pixel number) matrix, *emHDSS* is a 2,048×2 (channel number × number of fluorophores) matrix and *A* is a vector of 2 numbers representing the relative concentrations of the two fluorophores. The two endmembers of the unmixing problem are the HDSS of the two fluorophores concatenated in the endmember matrix *emHDSS.* These two endmembers were estimated from the synthetic data by the first and last row of the dataset (see Fig. 4a) where fractional abundances were known to be equal to 1 and 0.

### Figures of Merit for Accuracy and Precision

A figure of merit for accuracy (Fig. 4c) was defined as the average of the differences between the values of the fractional contributions of a reference fluorphore (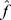) and its respective estimated value (*f*): 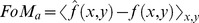. Each *x-*th column of the simulated gradients (Fig. 4a) contains 256 repeats of the same simulation; it was thus used to estimate the variance 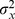 on the estimation of the fractional contribution. A figure of merit of precision (Fig. 4d) was therefore defined as the average standard deviation across the gradient normalized by Poissonian noise 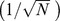: 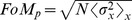. Where *N* is the total photon count per pixel and 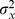 is defined by the following equation: 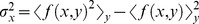.

### Proof of Photon Partitioning Theorem

Because fluorescence emission is a Poissonian process, if *G_i_* is the expected count, the probability *p_i_* to count *n_i_* photons in the *i*-th channel is:
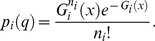
(2)


*G_i_* represents the expected photon counts in the *i*-th channel; *G_i_* is the integral of 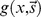, the photon-detection probability; *g* depends on various spectral coordinates (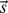) and the parameter *x*:
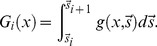
(3)


Here, *x* denotes the generic quantity we are interested to estimate. Therefore, the likelihood and the log-likelihood of the detection process over *m* independent channels are:
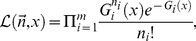
(4a)

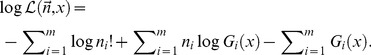
(4b)


Under basic assumptions of regularity and differentiability, the analytical representation of the Fisher information can be obtained computing the negative of the expectation of the second derivative of the log-likelihood that describes the experiment:
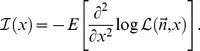
(5)


Functions that describe the fluorescence emission of fluorophores at room temperature are regular and smooth, thus this identity can be considered always true. Therefore, it is simple to show that:
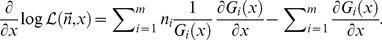
(6)


From this follows that:
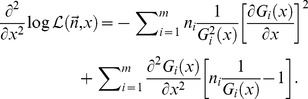
(7)


Because the expectation of 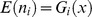, the Fisher information in fluorescence detection is described by Eq. (8):
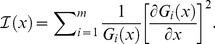
(8)


In order to show that the Fisher information increases with the addition of photon counting channels, let π’ be a finer partition of *N* photons relative to π. If we demonstrated that the partition of one channel into two independent noiseless channels generally result into an increase in Fisher information, then this will be true for more complex finer partitions. Let partition only one channel of the partition π into two independent noiseless channels (*i.e.*, *m*’ = *m*+1). It is also convenient to define the function 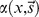, the photon partitioning function, that will describe the fraction of 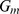 photons that will be counted in the new channel 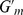:
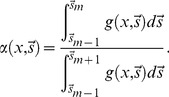
(9)


Therefore, the Fisher information carried by the two new detection channels will be equal to:
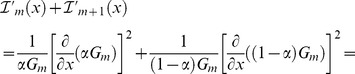


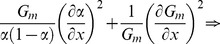


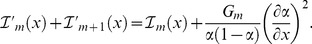
(10)


Therefore, inasmuch as the second addendum in Eq. (10) is always positive or null, we have proven that partitioning the *m*-th channel of π into two channels, the Fisher information theory increases or remain equal. This is applicable for any other *i*-th channel. Therefore, for any given finer partition, the Fisher information will be larger or equal to the Fisher information related to 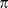:
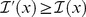
(11)


### Proof of the Non-trivial Partitioning Corollary

Theoretically, detection channels with different (hyper) spectral characteristics can be combined to generate a large number of possible partitions of photons collected in any given experiment. The trivial duplication of a channel will not increase the Fisher information. A finer partition 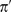 of *N* 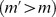 for which 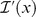 is strictly greater than 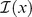 will be called *non-trivial* as it will increase the information content of the experiment on the unknown *x*. Provided a partitioning function 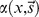 with values bound in (0,1) such that the two new detection channels will count 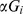 and 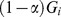, respectively, it is possible to infer the following necessary and sufficient condition from Eqs. (10):
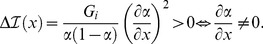
(12)


We note that the mere duplication of a channel is trivial by definition and exhibit null derivative of the partitioning function therefore resulting in no gain of information.

### Partitioning Function: An Example

Although the partitioning function has such a fundamental role in the optimization of detection schemes, we have demonstrated that optimization can be achieved without analytical solutions, but simply applying the general principles described by the photon partitioning theorem and its corollaries. However, it is instructive to analyze the partitioning functions for the simple case of fluorescence lifetime detection in the case that the random variable *x* is the fluorescence lifetime (τ) of the fluorophore. Let’s consider two time gates delimited by the relative times *t_1_*>*t_2_*>*t_3_*. In this case, the only spectral property considered for the partitioning is time and the vector 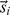 is simply a number equal to *t_2_*, the common boundary of the two gates. The photon counts in each time gates are simply the integrals of exponential functions and the partitioning function (*i.e.*, the fraction of photons detected within *t_1_* and *t_2_* over the total photons detected within *t_1_* and *t_3_*) is:
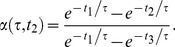
(13)


The derivative of 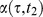 is clearly defined and continuous for 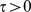 and *t*_3_>*t*_1_, conditions always true. In fluorescence detection at room temperature, spectra, anisotropy and lifetime decays are always represented by smooth and continue functions and, therefore, partitioning functions are always defined.

### Proof of Photon-efficiency Corollary

The Fisher information is the inverse of the Rao-Cramer limit on the variance of an unbiased estimator. It is therefore rather trivial to demonstrate that for any non-trivial partitioning the following inequality holds:
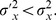
(14)


This implies that with an increasing number of independent non-trivial noiseless channels the estimate of the unknown will be measured with decreasing uncertainty. We can therefore generalize the concept of photon-economy (or photon-efficiency) that has been described for fluorescence lifetime imaging microscopy [2]. The signal-to-noise ratio with which is possible to estimate *x* increases with the non-trivial addition of detection channels. This can be described by the F-value, 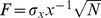, *i.e.* the ratio between the relative error 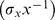 that affects the estimate of *x* compared to Poissonian noise 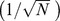. It is rather immediate to show that when 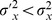, 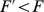. Furthermore, a definition of photon-efficiency is 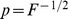 [3]. For instance, if *F* = 1, *p* = 1 and the SNR 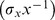 in *x* is the same defined by Poissonian noise 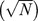; if *F* = 2, p = 0.25 and the SNR in x twice as large than 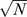. In order to recover the same SNR, four times the number of photon should be detectec and, therefore it is as if 75% of photons were lost (the photon-efficiency is equal to 0.25). Therefore, when 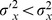, *p’* >*p*.

### Proof of Separability and Resolving Power Corollary

It is difficult to establish a general criterion for the resolution of biochemical techniques. In practical cases, however, biochemical systems are characterized by a parameter *x*. This parameter may be FRET efficiency, the fraction of interacting molecules, an enzymatic activity, etc. The distance between two biochemical systems can be therefore quantified by the Euclidian distance between the two values of *x*: 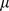 = |*x_1_*−*x_2_*|. We can thus define the separability of two biochemical signatures by:
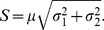
(15)


For non-trivial partitions of higher channel densities, both the uncertainties 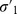 and 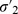 will be smaller or equal than σ_1_ and σ_2_, therefore, the separability of the detection system tend to increase, i.e., *S*’≥*S*. *S* can be used as a robust metric for the biochemical resolving power in fluorescence detection. However, it is possible to apply the typical criteria used to define the spatial resolution in microscopy. In the approximation of normal distributions, the Rayleigh criterion correspond to a separability *S* = 2 and with *N* photons measured for both *x_1_* and *x_2_* (assumption inherent in the Rayleigh criterion). Therefore, the smallest difference Δ*x* that can be resolved around the value *x* = *x*_0_ can be evaluated by substituting in Eq. (15) *x_1_*, *x_2_*_,_ σ_1_ = σ_2_ = σ_0_ (assumption inherent in the Rayleigh criterion) with *x_0_*+Δ*x*/2, *x_0_*–Δ*x*/2 and 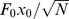, respectively, and solving for Δ*x*: 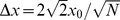. This definition can be written in a more elegant form, using the photon-efficiency *p*:
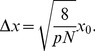
(16)


At the net of its spatial resolution, the biochemical/photophysical resolving power (*x_0_*/Δ*x*) of a fluorescence microscope is thus:
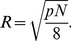
(17)


As we have demonstrated that for a non-trivial partitioning of channels increases the photon-efficiency of a detection system (*p’* >*p*), it implies that the non-trivial partitioning of channels improves the biochemical/photophysical resolution 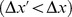 and resolving power (*R’*>*R*) of fluorescence microscopy. For the effect of the instrument response function of an instrument, see Text S2– Biochemical Resolving Power.

### Optimal Gates for Two Time Gates System: Analytical Solution

Li *et al.* have evaluated the analytical description of the F-value for a time gated system with two time gates of uneven width [13]. For simplicity, we will consider the analytical solution for two adjacent (non-overlapping) time gates only and redefine the parameters *x*, *S* and *R* in Eq. (2) in Li *et al.* [13] substituting 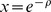, *S* = 0 *(i.e.*, non-overlapping gates*)*, 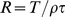 (*i.e.*, the second gate ends at the laser period *T* and starts at time 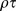). Eq. (2) in Li *et al.* [13] is already an approximated analytical solution (see appendix in [13]), but for further simplicity we can compute solutions for values of *T* large enough 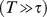 to avoid significant number of photons to be collected in subsequent laser pulses:
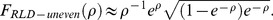
(18)


Therefore, by a simple derivation of Eq. (18), it is possible to prove that the best *(i.e.*, its minimum) value for 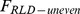 is 1.24 for 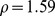.

### Optimal Gates for Two Time Gates System: Monte Carlo Simulations

Mono-exponential fluorescence decays with τ = 2 ns and 250, 1000 and 10000 photon/pixel were simulated, in the presence of Poissonian noise, to find the optimal configuration of a two time gates based system with uneven gate width. The time range between 0–50 ns was used in order to include all photons emitted by the simulated fluorophores. Simulated photon counts were binned into two gates and the F-value was estimated using 15,000 replicates of the simulation in order to estimate the precision of the technique. The beginning of the second time gate was scanned from 0 to 10 ns in 0.05 ns steps. The best F-value for all three photon-count levels was 1.24 at 1.59τ.

### Numerical Optimization of Fisher Information

In order to optimize Fisher information, it is possible to implement numerical iterative strategies that maximize the gain of Fisher information when creating a denser partition of detection channels. The Rao-Cramer lower bound for the precision of a measurement for a given partition is 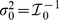. As the number of channels is increased, the new lower limit is 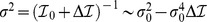, where the approximation is provided by the zeroth and first elements of the Taylor series; the gain of information 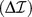 can be evaluated numerically by 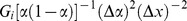. The addition of a channel may introduce noise 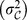: 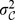 can be either considered a generic cost, a numerical tolerance or it can represent actual noise (*e.g.*, a photon-loss, read-out noise, dark counts) introduced when adding a detection channel. Therefore, iterative algorithms for the numerical optimization of partitions can impose the requirement 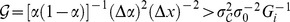 to allow a channel being partitioned into two new channels. 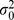 cannot be always evaluated, but its lower limit is necessarily Poissonian noise, *i.e. G_i_*. Therefore, it is convenient to apply a stronger constrain to the numerical optimization by using the condition 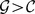 with 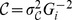. The interpretation of the cost is straightforward, but it will be different for different applications (see also **Material S1– Costs and photon-losses: case studies**). For instance, if a single photon counting system is used, with maximum photon count rate (MCR) and a dark count rate (DCR), 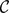 is equal to *(DCR/MCR)N* ^−*1*^ *,* where *N* is the total count measurable in a given amount of time. For a typical system with DCR∼1 kHz, MCR∼1 MHz and considering 100 photons counted, 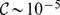. The numerical factor 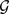 was defined for the case in which photons initially detected by one channel are then partitioned into two channels one counting a fraction of photons and the second counting the fraction 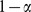. In this case, an iterative algorithm using the criterion 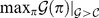 can be used. The same algorithms can be used to evaluate the loss of information 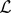 when merging two channels 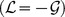, thus using the criterion 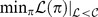 to search for the best detection system. The software provided in Supplementary Material relies on the numerical maximization Fisher information by the analysis of 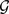 for time gating (Fig. 1) and by the analysis of 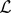 for spectral gating in order to offer examples and useful tools to optimize FLIM and spectral imaging with these bottom-up (increasing channel number) and top-down (decreasing channel number) strategies. Fisher information was evaluated numerically using directly Eq. (8). Substituting *G_i_* with *f_i_N*, where *f_i_* is the fraction of total photon number (*N*) detected by the *i*-th channel, it is simple to show that the F-value can be evaluated numerically and minimized using the factor 
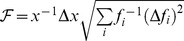
. Optimization strategies with the direct minimization of 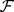 would be computationally expensive. Therefore, a second iterative algorithm that minimizes 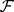 was used only when the optimal number of detection channels was found maximizing 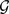 or 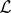 by the use of the criterion 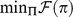. Contrary to the direct application of the photon partitioning theorem and its corollaries, the optimization of 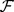 is not always straightforward.

For instance, in the case of spectral imaging, the relative fraction of one fluorophore depends on the abundances of both fluorophores. In this case, the Fisher information is a matrix and its numerical evaluation can be carried out as:
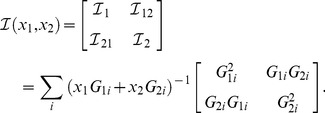
(19)


Inverting the Fisher information matrix permits to find the covariance matrix:
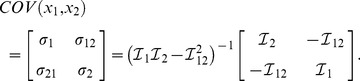
(20)


Many figure of merits can be defined to evaluate the combined error on the estimation of the relative abundances; for the sake of example, the software we provide uses the average of relative errors:
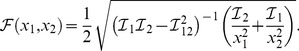
(21)


## Supporting Information

Figure S1 Spectral unmixing of spectrally overlapping fluorescent proteins. Spectral unmixing on images of cells expressing EGFP-Actin and EYFP-Tubulin is described in the main text and in Figure 4.
(DOCX)

Figure S2 Hyper Dimensional Spectral Signatures of two synthetic fluorophores peaked at 540 nm, 560 nm and their sum. These HDSS have been used to test unmixing algorithms (see Figs. 4 and 5).
(DOCX)

Text S1 Costs and photon-losses: case studies. A review of photon losses in optical relay systems.
(DOCX)

Text S2 Biochemical Resolving Power. The definition of biochemical resolving power.
(DOCX)

Text S3 Hyper Dimensional Phasors (HDPH). The definition of phasors used to analyse the data.
(DOCX)

Material S1 Matlab code for Fisher information analysis. The zipped folders contain three subfolders with software designed to optimize Fisher information numerically, making use of the photon partitioning theorem and its corollaries: • *fpt_flim_fig1* provides examples of optimization of time gates for FLIM. This software was used to generate some of the panels in Fig. 1. Run *fpt_optimize_time_gates.m* to find the best partition. • *fpt_spec_fig2* provides examples of optimization of spectral gates for spectral imaging. This software was used to generate some of the panels in Fig. 2. Run *fpt_optimize_spectral_gates.m* to find the best partition. • *fpt_check_dimensionality_fig5* provides example of optimization for a generic system. It accepts two HDSSs as an input and it will provide Fisher information analysis for systems capable of fluorescence lifetime, spectral and anisotropy detection and all combinations of these techniques. Run *fpt_check_dimensionality.m* to find the best detection system to separate two optical signatures. This code was used to generate Fig. 5.
(ZIP)
