# Supplementary material for: Maximizing the Biochemical Resolving Power of Fluorescence Microscopy
Source: PLoS One. 2013 Oct 28;8(10):e77392. doi: 10.1371/journal.pone.0077392 (PMC3810478; doi:10.1371/journal.pone.0077392)
Supplement: Figure S1 — Spectral unmixing of spectrally overlapping fluorescent proteins. Spectral unmixing on images of cells expressing EGFP-Actin and EYFP-Tubulin is described in the main text and in Figure 4. (DOCX) [file pone.0077392.s001.docx]

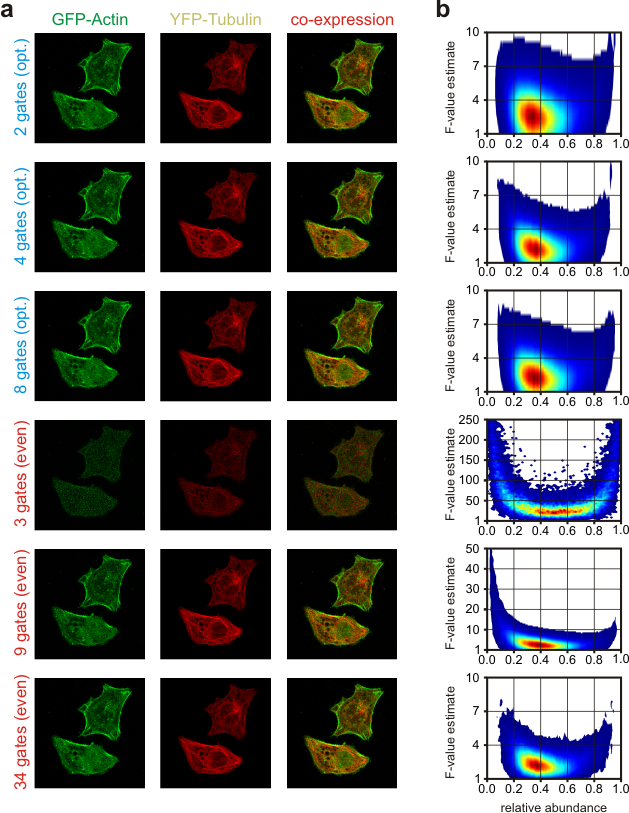


**Supporting Figure S1 | Spectral unmixing of spectrally overlapping fluorescent proteins.** Spectral unmixing on images of cells expressing EGFP-Actin and EYFP-Tubulin is described in the main text and in Fig. 3. Here, the relative abundance of EGFP-Actin (**a**, left column) and EYFP-Tubulin (**a**, middle column) are shown not only by an overlay (a, right column) in order to permit assessment of the quality of individual EGFP/EYFP signals. The distribution of F-values (see also Fig. 5) measured experimentally (**b**) on these images show that at higher channel number, spectral imaging exhibits higher precision. However, spectral channels optimized by the analysis of Fisher information (top three rows), provides excellent results also at low channel numbers compared to standard even partitions (bottom three rows).
