# Supplementary material for: Maximizing the Biochemical Resolving Power of Fluorescence Microscopy
Source: PLoS One. 2013 Oct 28;8(10):e77392. doi: 10.1371/journal.pone.0077392 (PMC3810478; doi:10.1371/journal.pone.0077392)
Supplement: Figure S2 — Hyper Dimensional Spectral Signatures of two synthetic fluorophores peaked at 540 nm, 560 nm and their sum. These HDSS have been used to test unmixing algorithms (see Figs. 4 and 5). (DOCX) [file pone.0077392.s002.docx]

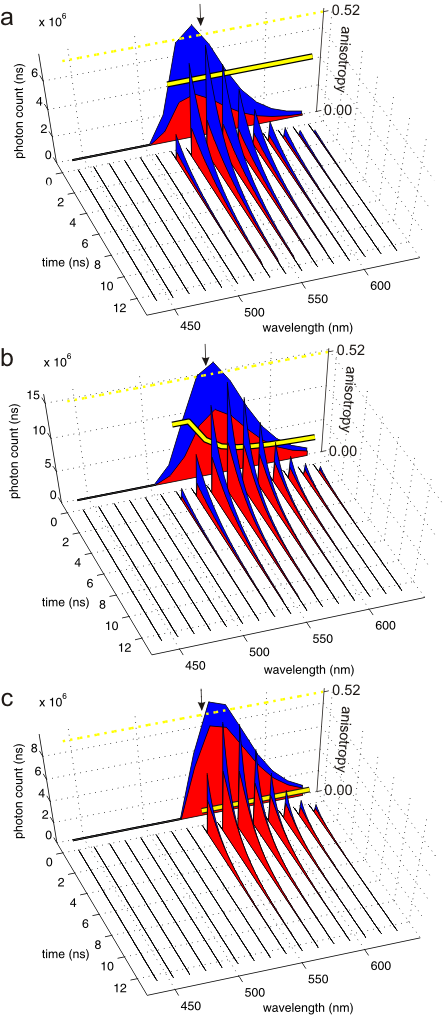


**Supporting Figure S2 | Hyper Dimensional Spectral Signatures of two synthetic fluorophores peaked at 540nm (a) and 560nm (c) and their sum (b).** Red and blue areas represent the perpendicular and parallel components of the fluorescence decays and emission spectra projections. Secondary vertical axis shows steady-state anisotropy projections depicted by the solid yellow lines. Dashed yellow line mark the maximum anisotropy value measurable with two photon excitation. These complex signatures fully define the emission of fluorophores. These HDSS have been used to test unmixing algorithms (see Figs. 4 and 5). Simulations have been carried out by varying the spectral shifts between the peaks of the two fluorophores around 550nm (see arrow).
