# Supplementary material for: Maximizing the Biochemical Resolving Power of Fluorescence Microscopy
Source: PLoS One. 2013 Oct 28;8(10):e77392. doi: 10.1371/journal.pone.0077392 (PMC3810478; doi:10.1371/journal.pone.0077392)
Supplement: Text S1 — Costs and photon-losses: case studies. A review of photon losses in optical relay systems. (DOCX) [file pone.0077392.s003.docx]

**Supporting Text S1 - Costs and photon-losses: case studies.** The main aim of this work is to define the physical limit of the biochemical resolving power in fluorescence microscopy. However, engineering a system with many detection channels, multi-parametric or not, poses several challenges. The quantitative detection of biochemistry often makes the use of multiple detection channels necessary: for instance, the detection of oligomerization by homoFRET requires two channels for polarization imaging; the detection of protein-protein interactions by heteroFRET requires either one time-resolved channel (in this work and the related literature a time-resolved channel is modelled with a plurality of independent gated photon counting channels) or at least two spectrally distinct channels; the unmixing of fluorophores requires a number of spectral channels. In all the aforementioned cases, the analysis of photon-losses *per se* is irrelevant inasmuch as the introduction of a number of channels is strictly necessary. However, if one dissected more carefully, for instance, a physical phenomenon such as FRET, it would be immediately apparent that FRET simultaneously alters the fluorescence lifetime, the colour and the anisotropy of the emission of a FRET pair (*e.g.*, EGFP and mCherry). The direct implication of the *photon partitioning theorem* is that multiple (parallel) detection channels detecting the various properties of light (*e.g.*, HDIM) results in higher precision and, consequently, a better (biochemical) resolving power of the microscope. Therefore, the analysis of photon-losses is fundamental to address the following questions. How many channels should be added to achieve optimal performance? How many parameters (polarization, wavelength, time) should be measured? In other words, which cost/benefit analysis should be performed in the optimization of the detection system? We have provided mathematical tools and algorithms to permit this analysis; however, managing photon-losses is so fundamental in the construction of a fluorescence microscope that we will illustrate our viewpoint using some realistic scenarios:

Spectral imaging. Multi-colour imaging can be achieved with the use of dichroic mirrors and filters. Even with the use of the best and steepest optical filters with peak transmittance close to 100%, there will be always optical losses. For instance, just to image EGFP and mCherry onto two different optical channels, it can be easily estimated from specifications of (the best) filter sets (500-540nm and 600-660nm bandpass filters) that ~40% and ~30% of the fluorescence emission spectra is lost, respectively. This is because not all available fluorescence emission spectrum is detected when using a filter as the separation of fluorophore is attempted by cutting light in hardware contrary to spectral systems that acquire all available photons and separate them by software. The best dichroic beamsplitter will also cause losses of ~10% in transmission for EGFP and ~5% in reflection for mCherry. Furthermore, 80% or 60% of light is lost because of the poor quantum efficiency of PMTs (multialkali and GaAsP photocathodes, respectively). Therefore, even the simplest detection system for two channels may lose at least 80-90% and 70-80% of the detectable photons for EGFP and mCherry, respectively. These losses should not be neglected when comparing single channel system to hyperspectral detection.

In order to estimate optical losses in dispersive systems (*e.g.*, the Leica SP5), we analysed losses in a custom built optical relay using a small dispersive prism with standard anti-reflection coating and a camera lens (See [[1](#_ENREF_1)] for details). With the use of a supercontinuum light source with a 400-600nm band pass filter, a power loss from 49.1±0.2 mW to 39.0±0.1 mW was measured across the prism and camera lens (~20% photon-loss). For hyperspectral imaging, a multi-anode PMT (either with multialkali or GaAsP photocathode) can be used for detection, at the cost of an additional ~20% optical loss caused by the masks placed within anodes if used without a microlens array. Therefore, a hyperspectral detection system exhibits a photon-loss of 74-87% depending on the sensitivity of the photocathode. This loss is comparable to the losses incurred with filter based systems (74-87% *versus* 70-90%) but the simultaneous measurement of 16-32 spectral channels results in higher signal to noise ratios in spectral unmixing (see Theory section). The same spectral sampling can be achieved with sequential imaging for adjacent spectral bands; however, for each acquisition the large majority of photons are not collected. For instance, 16 spectral acquisitions incur to a net loss of 94% of light to be compounded with losses incurred at the detector.

Moreover, nowadays spectrographs built for confocal/two-photon microscopy can be built around a dispersive prism in combination with an EM-CCDs for the detection [[1](#_ENREF_1),[2](#_ENREF_2)]. The high quantum efficiency of an EM-CCD (>90%) and the low losses of dispersive prisms (~20%) permit the reduction of photon-losses down to ~30% (compared to >70% losses of other systems), yet providing up to 128 spectral windows [[1](#_ENREF_1),[2](#_ENREF_2)]. This value is likely to be further improved with the use of customized prisms and anti-reflection coatings. Therefore, hyperspectral detection can provide parallel detection channels and lower photon-losses (from 70-90% down to <30%).

Anisotropy imaging. Fluorescence anisotropy imaging relies on the use of at least two polarization-dependent detectors. These can be operated sequentially or in parallel, for instance using a polarizer beam splitter. When used in parallel, an optical loss of ~5% in transmission and a negligible loss (<1%) in reflection are typical. These losses are comparatively low, but they may compound losses caused by linear polarizers often used to increase the contrast ratio of polarizer beam splitters potentially causing a total loss of 70% of light. When the two polarization states are acquired sequentially, an analyser of similar efficiency is used but light is also lost during the two sequential exposures (50%) bringing the total losses of light to ~85%. Therefore, when considering also detection with a typical PMT, the photon-losses in anisotropy imaging can reach 90-95% (parallel detection) or 94-97% (sequential detection) depending on the sensitivity of photocathodes. However, we have recently implemented a method to orthogonally disperse light depending on both polarization and wavelength using a relay system made of a Wollaston prism, a dispersive prism and a camera lens [[1](#_ENREF_1)]. We measured a drop of power of a supercontinuum source filtered between 400-600nm with a bandpass filter, from 57.8±0.1mW to 49.1±0.1mW measured before and after the Wollaston prism corresponding to a loss of ~15% of light. Together with the rest of the relay system (prism and lens) and an EM-CCD camera, this spectropolarimeter characterizes the emission spectrum and polarization state at each wavelength on 2x128 spectral windows with a total loss of less than 40% of light. This loss can be further reduced with the construction of dedicated cemented optical systems and optimized coatings.

Fluorescence lifetime imaging. The parallel detection of time-gates is done routinely with laser scanning microscopes and the addition of time-gates does not result in any loss. Therefore, it is mostly relevant to discuss extension of FLIM to spectral detection or HDIM in a similar way we have described for other spectrally resolved techniques. However, in *Methods - Numerical optimization of Fisher information* we also describe how the “cost” for the addition of a (time) channel can be also additional Dark Noise rather than a photon-loss. Here, we focus on photon-losses and the analysis of detection of FRET (e.g, EGFP and mCherrry exhibiting a FRET efficiency of 30%) is very instructive. With a single channel FLIM system, it is possible to detect 20-40% of the photons reaching the detector (again depending on the sensitivity of the photocatode) after a loss incurred with the use of an EGFP filterset (~40% as described earlier) and a loss of 30% of photons caused by FRET, resulting into a total loss of 83-92%. With the use of a dichroic beamsplitter and a filter for mCherry, the 30% of signal lost by FRET can be partially recovered at the net of a loss (70-80%) caused by the filters for the acceptor channel. Therefore, this second detection channel diminishes the total losses to 75-87% (from 83-92% of a single channel system). This is one example where a multi-channel detection allows both to increase the Fisher information (about FRET) and to decrease photon-losses. Current spectral detectors based on arrays of PMTs capable of time-resolved applications exhibit still a low quantum efficiency (15%) and lose 20% of light in the masks of the multi-anode arrays. By trading optical losses of filters with a 20% loss of a dispersive prism, a spectrally resolved FLIM can exceed losses of 90%. Although high, this value is within the range of single channel systems (83-92%) and, at the same time, spectrally resolved FLIM provides significantly higher Fisher information content. It should be also noted that new detector technologies are or will be soon commercially available: PMT arrays with GaAsP photocathodes (>40% quantum efficiency with 80% fill-factor), linear arrays of SPADs or silicon photomultipliers (SiPM, >32% quantum efficiencies and ~34% fill-factor) [[3](#_ENREF_3),[4](#_ENREF_4)], and directly modulated EM-CCDs (~40% quantum efficiency and 44% fill factor) [[5](#_ENREF_5)]. In combination with a spectropolarimeter, these technologies will result in total losses of 78%, 93%, 88%. These values are not just in the range of single channel systems, but they will provide the complete characterization of the photophysics of the samples providing very high information content with HDIM. Moreover, the latter two technologies are state-of-the-art systems that still need to reach production. SPADs or SiPMs are capable to reach quantum efficiencies in excess of 60% with ~90% fill-factors with microlens arrays. Modulated EM-CCDs, should be able to reach quantum efficiencies of 90% with a fill factor ~50% (currently limited by the presence of in-pixel multi-storage areas).

Conclusion. This overview about photon-losses in optical relay systems for multi-channel / multi-parametric imaging microscopy demonstrates that the analysis of photon-losses is fundamental when engineering a detection system; however, it also emphasizes that a multi-channel / multi-parametric system can be engineered in order also to minimize photon-losses in particular when used together with dispersive photonic devices of high transmittance thanks to the parallelization of the imaging process.

1. Esposito A, Bader AN, Schlachter SC, van den Heuvel DJ, Schierle GS, et al. (2011) Design and application of a confocal microscope for spectrally resolved anisotropy imaging. Opt Express 19: 2546-2555.

2. Palero JA, de Bruijn HS, van der Ploeg-van den Heuvel A, Sterenborg HJCM, Gerritsen HC (2006) In vivo nonlinear spectral imaging in mouse skin. Optics Express 14: 4395-4402.

3. Pancheri L, Stoppa D (2009) A SPAD-based Pixel Linear Array for High-Speed Time-Gated Fluorescence Lifetime Imaging. 2009 Proceedings of Esscirc: 429-432.

4. Kiwanuka SS, Laurila TK, Frank JH, Esposito A, Blomberg von der Geest K, et al. (2012) Development of broadband cavity ring-down spectroscopy for biomedical diagnostics of liquid analytes. Anal Chem 84: 5489-5493.

5. Zhao Q, Schelen B, Schouten R, van den Oever R, Leenen R, et al. (2012) Modulated electron-multiplied fluorescence lifetime imaging microscope: all-solid-state camera for fluorescence lifetime imaging. J Biomed Opt 17: 126020.
