# Supplementary material for: Maximizing the Biochemical Resolving Power of Fluorescence Microscopy
Source: PLoS One. 2013 Oct 28;8(10):e77392. doi: 10.1371/journal.pone.0077392 (PMC3810478; doi:10.1371/journal.pone.0077392)
Supplement: Text S2 — Biochemical Resolving Power. The definition of biochemical resolving power. (DOCX) [file pone.0077392.s004.docx]

**Supporting Text S2 – Biochemical Resolving Power.** The aim of this work is to define the biochemical resolution of a microscope and its theoretical limit at the net of technological implementations. Therefore, the description of the *instrument response function* (IRF) was omitted from the main text of the manuscript. For completeness, we report here the effect of an ideal Gaussian IRF on biochemical separability, resolution and resolving power:

$S=\mu\sqrt{\sigma_{1}^{2}+\sigma_{2}^{2}+2\sigma_{IRF}^{2}}$,

,

.

In this case, in the presence of very high SNR (*N*🡪∞) all these figures converge to values defined by the instrument response function and a constant determined by the Rayleigh criterion.
