# Supplementary material for: Maximizing the Biochemical Resolving Power of Fluorescence Microscopy
Source: PLoS One. 2013 Oct 28;8(10):e77392. doi: 10.1371/journal.pone.0077392 (PMC3810478; doi:10.1371/journal.pone.0077392)
Supplement: Text S3 — Hyper Dimensional Phasors (HDPH). The definition of phasors used to analyse the data. (DOCX) [file pone.0077392.s005.docx]

**Supporting Text S3 – Hyper Dimensional Phasors (HDPH).** Phasors are becoming very popular for the analysis of fluorescence images because they provide a model-free and graphical interpretation and visualization of the data. Phasor transforms have been defined for time-resolved, spectrally-resolved and polarization-resolved images. Here we combine these definitions and extend them to multi-dimensional data therefore providing multi-parametric and novel multi-dimensional phasor transforms. The equations below are the definition of the 16 phasor transforms used for the data reduction algorithm described in the manuscript. These 16 numbers per pixel were used to compress HDIM information (from 2,048 numbers/pixel) and for subsequent spectral unmixing.

Definition of constants and variables:

**DCT** digital cosine transform

**DST** digital sine transform

**I_tot_** total photon count per pixel

**x,y** pixel location

**g** index of the time gate

**c** index of the spectral gate

**t_g_** average arrival time of the time gate **g**

**T** period of the laser pulse

**λ_c_** average wavelength of the spectral gate **c**

**∆λ** total spectral bandwidth

**I_⊥_** photon counts detected in the orthogonal polarization component

**I_||_** photon counts detected in the parallell polarization component
